# Blood pressure variability is related to faster cognitive decline in ischemic stroke patients: PICASSO subanalysis

**DOI:** 10.1038/s41598-021-83945-z

**Published:** 2021-03-03

**Authors:** Yerim Kim, Jae-Sung Lim, Mi Sun Oh, Kyung-Ho Yu, Ji Sung Lee, Jong-Ho Park, Yong-Jae Kim, Joung-Ho Rha, Yang-Ha Hwang, Sung Hyuk Heo, Seong Hwan Ahn, Ju-Hun Lee, Sun U. Kwon

**Affiliations:** 1 Department of Neurology, Kangdong Sacred Heart Hospital, Hallym University College of Medicine, Seoul, Republic of Korea; 2 Department of Neurology, University of Ulsan, Asan Medical Center, Seoul, Republic of Korea; 3 Department of Neurology, Hallym University Sacred Heart Hospital, Hallym University College of Medicine, Anyang, Republic of Korea; 4 Clinical Research Center, Asan Institute for Life Sciences, Asan Medical Center, University of Ulsan College of Medicine, Seoul, Republic of Korea; 5 Department of Neurology, Myongji Hospital, Hanyang University College of Medicine, Goyang, Republic of Korea; 6 Department of Neurology, Eunpyeong St. Mary’s Hospital, College of Medicine, The Catholic University of Korea, Seoul, Republic of Korea; 7 Department of Neurology, Inha University Hospital, Incheon, Republic of Korea; 8 Department of Neurology, Kyungpook National University Hospital, School of Medicine, Kyungpook National University, Daegu, Republic of Korea; 9 Department of Neurology, Kyung Hee University Medical Center, Seoul, Republic of Korea; 10 Department of Neurology, Chosun University Hospital, Gwangju, Republic of Korea

**Keywords:** Neuroscience, Medical research, Neurology

## Abstract

Blood pressure variability (BPV) is associated with higher cardiovascular morbidity risks; however, its association with cognitive decline remains unclear. We investigated whether higher BPV is associated with faster declines in cognitive function in ischemic stroke (IS) patients. Cognitive function was evaluated between April 2010 and August 2015 using the Mini-mental State Examination (MMSE) and Montreal Cognitive Assessment in 1,240 Korean PICASSO participants. Patients for whom baseline and follow-up cognitive test results and at least five valid BP readings were available were included. A restricted maximum likelihood–based Mixed Model for Repeated Measures was used to compare changes in cognitive function over time. Among a total of 746 participants (64.6 ± 10.8 years; 35.9% female). Baseline mean-MMSE score was 24.9 ± 4.7. The median number of BP readings was 11. During a mean follow-up of 2.6 years, mean baseline and last follow-up MMSE scores were 25.4 ± 4.8 vs. 27.8 ± 4.4 (the lowest BPV group) and 23.9 ± 5.2 vs. 23.2 ± 5.9 (the highest BPV group). After adjusting for multiple variables, higher BPV was independently associated with faster cognitive decline over time. However, no significant intergroup difference in cognitive changes associated with mean systolic BP was observed. Further research is needed to elucidate how BPV might affect cognitive function.

## Introduction

Stroke survivors often experience cognitive impairment^1^, and post-stroke dementia is associated with poor outcomes^2^. Approximately 65% of stroke survivors experience cognitive decline, with 30% developing dementia^1^. Some demographic factors, including increased age, lower education level^3^, and stroke-related factors including hemiplegia, pre-stroke antiplatelet use^4^, and imaging factors including white matter hyperintensities (WMHs) and medial temporal lobe atrophy^5^, are associated with cognitive decline after stroke. Baseline cognitive impairment before stroke^2^ is an important predictor for post-stroke dementia^6,7^.

Blood pressure variability (BPV), the normal oscillation of blood pressure (BP), is associated with higher risks of cardiovascular events^8^ and morbidity^9^. Additionally, previous studies^10–17^ demonstrated an association between high BPV and low cognition in various populations, although a causal relationship has not been established. A possible hypothesis is that BP fluctuations provoke ischemic damage to the white matter through cerebral hypoperfusion^18^. Carotid astherosclerosis^19^ and increased large arterial stiffness might reduce baroreceptor sensitivity and contribute to cerebral damage^20^. Considering that stroke patients’ auto-regulatory capacities are already impaired, making them more vulnerable to BP fluctuations, a high BPV might be a predictive factor for post-stroke cognitive decline.

Since cognitive function varies with time after the index stroke, physicians should evaluate cognitive outcomes serially rather than at a single point. Regarding the methodological aspect of cognitive evaluation, prior studies were limited in that they did not reflect cognitive changes over multiple visits in a longitudinal follow-up study^10,12,14^.

Therefore, in this study, we sought to determine whether BPV affects cognitive decline in patients with acute ischemic stroke (IS) by assessing BPV during each visit interaction. We hypothesized that a higher visit-to-visit BPV is associated with a faster decline in cognitive function in IS patients.

## Results

### Baseline characteristics

A total of 746 participants were included in the MMSE analysis (mean age, 64.6 ± 10.8 years; 35.9% female). Included patients were younger (64.6 ± 10.8 versus 67.8 ± 10.4 years). The mean education length was 8.6 ± 4.8 years. Baseline stroke severity was milder (NIHSS 1.8 ± 2.0 versus 2.6 ± 3.0). The baseline characteristics for participants included in and excluded from the analysis are shown in Table S1.

Among the included participants, initial median NIHSS score was 1 (interquartile range [IQR], 0–3). The mean MMSE score was 24.9 ± 4.7. The mean follow-up period was 2.6 years (IQR, 2.0–3.1), while the mean number of BP readings was 11 (IQR, 8–14).

Detailed baseline characteristics were stratified according to BPV-SD (Table 1). Compared with patients in the T1 group, patients in higher tertile groups were older and had fewer education years, lower cognitive function, and higher mean baseline BP.

**Table 1:** Baseline characteristics according to tertile of blood pressure variability using standard deviation.

	T1	T2	T3	*p*-value
Number of subjects	248	249	249	
Age, years (SD)	62.6 ± 10.8	64.2 ± 10.4	67.0 ± 10.8	< .0001
Sex, women, n (%)	86 (34.7)	81 (32.5)	101 (40.6)	0.1541
Education, years	9.2 ± 5.0	9.0 ± 4.5	7.6 ± 4.9	0.0002
Hypertension, n (%)	212 (85.5)	222 (89.2)	234 (94.0)	0.0081
Diabetes, n (%)	73 (29.4)	78 (31.3)	80 (32.1)	0.8008
Hyperlipidemia, n (%)	116 (46.8)	105 (42.2)	101 (40.6)	0.3490
Use of lipid lowering agent	197 (79.4)	187 (75.1)	181 (72.7)	0.2061
Coronary artery disease, n (%)	12 (4.8)	8 (3.2)	11 (4.4)	0.6411
Atrial fibrillation, n (%)	0 (0.0)	0 (0.0)	0 (0.0)	–
Smoking, n (%)	119 (48.0)	123 (49.4)	113 (45.4)	0.6608
**Index event, n (%)**				0.2113
Ischemic stroke	232 (93.5)	241 (96.8)	234 (94.0)	
Transient ischemic attack	16 (6.5)	8 (3.2)	15 (6.0)	
**Index of high risk of ICH**				0.3124
Prior history of ICH	41 (16.5)	42 (16.9)	38 (15.3)	
Imaging findings of ICH without clinical history	48 (19.4)	35 (14.1)	53 (21.3)	
Multiple microbleeds	159 (64.1)	172 (69.1)	158 (63.5)	
Baseline median NIHSS (IQR)	1 (0–3)	1 (0–3)	1 (0–3)	0.6002
**Baseline median MMSE (IQR)**	27 (24–29)	26 (23–28)	25 (21–28)	0.0007
24 or less, n (%)	72 (29.0)	79 (31.7)	110 (44.2)	0.0008
> 24, n (%)	176 (71.0)	170 (68.3)	139 (55.8)	
Baseline median MoCA (IQR)	21 (17–25)	21 (16–24)	19 (14–23)	< 0.0001
**Treatment**				
Cilostazol, n (%)	123 (49.6)	125 (50.2)	133 (53.4)	0.6577
Probucol, n (%)	126 (50.8)	122 (49.0)	127 (51.0)	0.8852
Mean SBP, mmHg (SD)	129.5 ± 13.1	131.8 ± 15.7	142.7 ± 22.6	< 0.0001
Mean DBP, mmHg (SD)	77.5 ± 10.1	77.8 ± 9.8	83.1 ± 14.7	0.0017
Median BP readings, (IQR)	10 (8–13)	13 (9–14)	11 (7–14)	0.0010
Median follow up periods, (IQR), unit: years	2 (2–3)	3 (2–4)	3 (2–3)	0.0022
**Baseline WMH, n (%)**				0.2694
Mild (Fazeka score = 1)	80 (33.9)	69 (29.1)	71 (29.2)	
Moderate to severe (Fazeka score = 2, 3)	156 (66.1)	168 (70.9)	172 (70.8)	

P-value by Chi-square test, ANOVA or Kruskal–Wallis test as appropriate.

*SD* standard deviation; *NIHSS* National Institutes of Health Stroke Scale; *MMSE* Mini-mental state examination; *MoCA* Montreal Cognitive Assessment; *IQR* interquartile range; *SBP* systolic blood pressure; *DBP* diastolic blood pressure; *WMH* whitematter hyperintensity; *ICH* intracerebral hemorrhage.

### Trajectories of cognition test scores over follow-up period

#### Analysis I: BPV and cognition test scores

**Figure 1:**
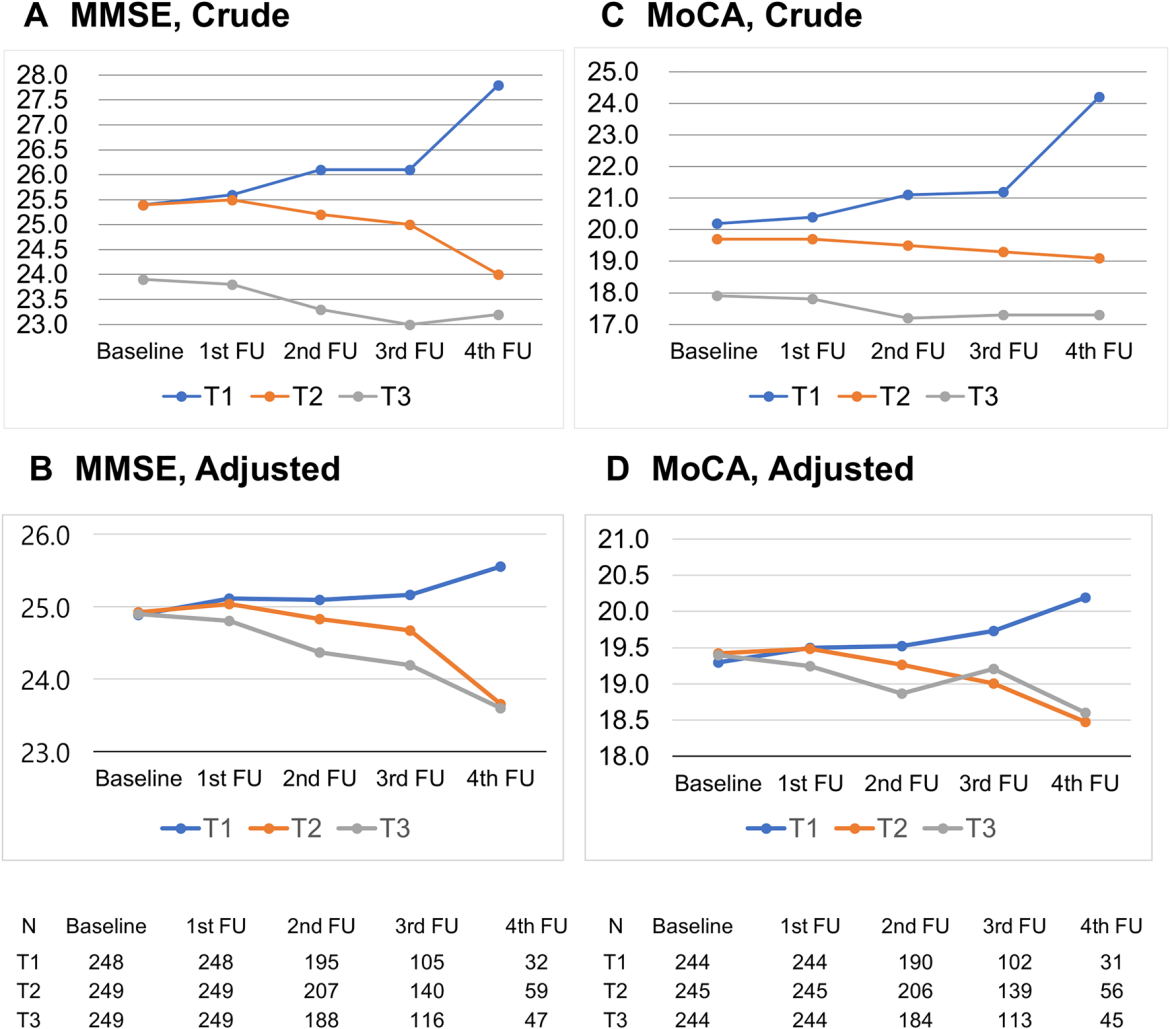
The cognition test scores from the Mixed-Model Repeated Measures analysis over the follow-up period according to the BPV-SD group. (**A**) MMSE, Crude (**B**) MMSE, Adjusted (**C**) MoCA, Crude (**D**) MoCA, Adjusted. *BPV-SD* intra-individual standard deviation of systolic blood pressure, *MMSE* Mini-mental State Examination, *MoCA* Montreal Cognitive Assessment.

We adjusted for age, sex, educational years, probucol treatment, baseline NIHSS score, baseline cognition test scores, diabetes and index of high risk of ICH, and mean SBP. Variables with p < 0.1 in univariate analysis or biological relevance were included in the multivariable model. During a mean follow-up of 2.6 years, MMSE scores from baseline to last follow up were 25.4 ± 4.8 vs. 27.8 ± 4.4 for the lowest BPV group, and 23.9 ± 5.2 vs. 23.2 ± 5.9 for the highest BPV group. The MMRM analysis showed that the cognition test scores over the follow-up period according to the BPV-SD group decreased rapidly. The trend using MMRM including BPV by visit interaction was statistically significant (*p* < 0.05) (Table 2). After adjusting for baseline cognitive status, linear trends of cognition test scores over the follow-up period were also significant across the groups (*p* for trend < 0.001) (Fig. 1A–D). The results of analysis using secondary BPV parameters (SDreg and VIM) were similar to those using BPV-SD, at least in the analysis using MMSE (Tables S2 and S3).

**Table 2:** Cognitive scores over visits according to tertile of BPV (SBP-SD) by using K-MMSE and K-MoCA.

	BPV Tertile 1 (ref.)	BPV Tertile 2	BPV Tertile 3	p-value^a^	p-value^b^	p-value^c^
**MMSE**						
Baseline	25.4 ± 4.8	25.4 ± 3.9	23.9 ± 5.2	0.0019	< .0001	0.0053
1st follow-up	25.6 ± 4.7	25.5 ± 4.2	23.8 ± 5.2			
2nd follow-up	26.1 ± 4.4	25.2 ± 4.4	23.3 ± 5.8			
3rd follow-up	26.1 ± 4.4	25.0 ± 5.0	23.0 ± 6.1			
4th follow-up	27.8 ± 4.4	24.0 ± 5.0	23.2 ± 5.9			
P for linear trend	0.1039	< .0001	< .0001			
**MoCA**						
Baseline	20.2 ± 6.4	19.7 ± 5.8	17.9 ± 6.4	0.0346	0.0007	0.4793
1st follow-up	20.4 ± 6.7	19.7 ± 5.9	17.8 ± 6.7			
2nd follow-up	21.1 ± 6.3	19.5 ± 6.3	17.2 ± 6.9			
3rd follow-up	21.2 ± 6.6	19.3 ± 6.6	17.3 ± 7.0			
4th follow-up	24.2 ± 5.4	19.1 ± 6.2	17.3 ± 8.1			
P for linear trend	0.0430	0.0025	0.0568			

Note. The Model included BPV group, visit, BPV-by-visit interaction, age, sex, educational year, probucol treatment, baseline NIHSS, baseline MMSE or MoCA, diabetes, index of high risk of intracerebral hemorrhage and mean SBP as fixed effect.

Effect of sites was adjusted as random in the model.

MMRM including BPV-by visit interaction, baseline score, and baseline score-by visit interaction.

*SD* standard deviation; *MMSE* mini-mental state examination; *MoCA* montreal cognitive assessment; *MMRM* mixed-model repeated measures.

^a^p-value by MMRM for BPV-by-visit interaction.

^b^p-value by MMRM for BPV effect.

^c^p-value by MMRM for visit effect.

#### Analysis II: Mean SBP and cognition test scores

**Figure 2:**
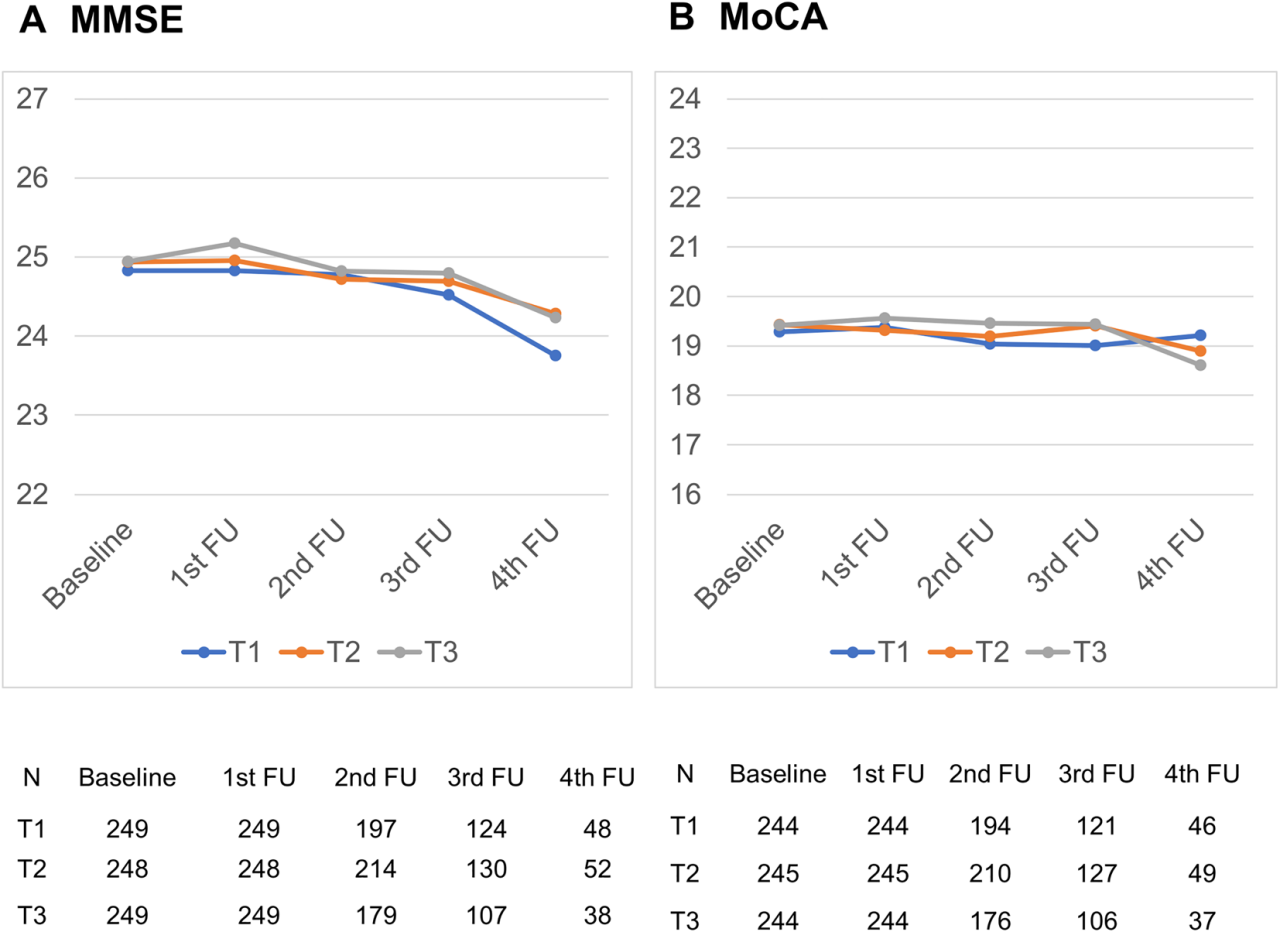
The cognition test scores from the Mixed-Model Repeated Measures analysis over the follow-up period according to the systolic blood pressure group. (**A**) Mini-mental State Examination (MMSE) (**B**) Montreal Cognitive Assessment (MoCA).

The detailed baseline characteristics between the tertile groups stratified according to mean SBP are presented in Table S4. After adjusting for multiple co-variables, MMRM analysis showed that the temporal cognitive changes over the follow-up period according to mean SBP were not statistically significant for the MMSE or MoCA test (Fig. 2A,B and Table S5).

#### Analysis III: Subgroup analysis stratified by baseline cognitive status

**Figure 3:**
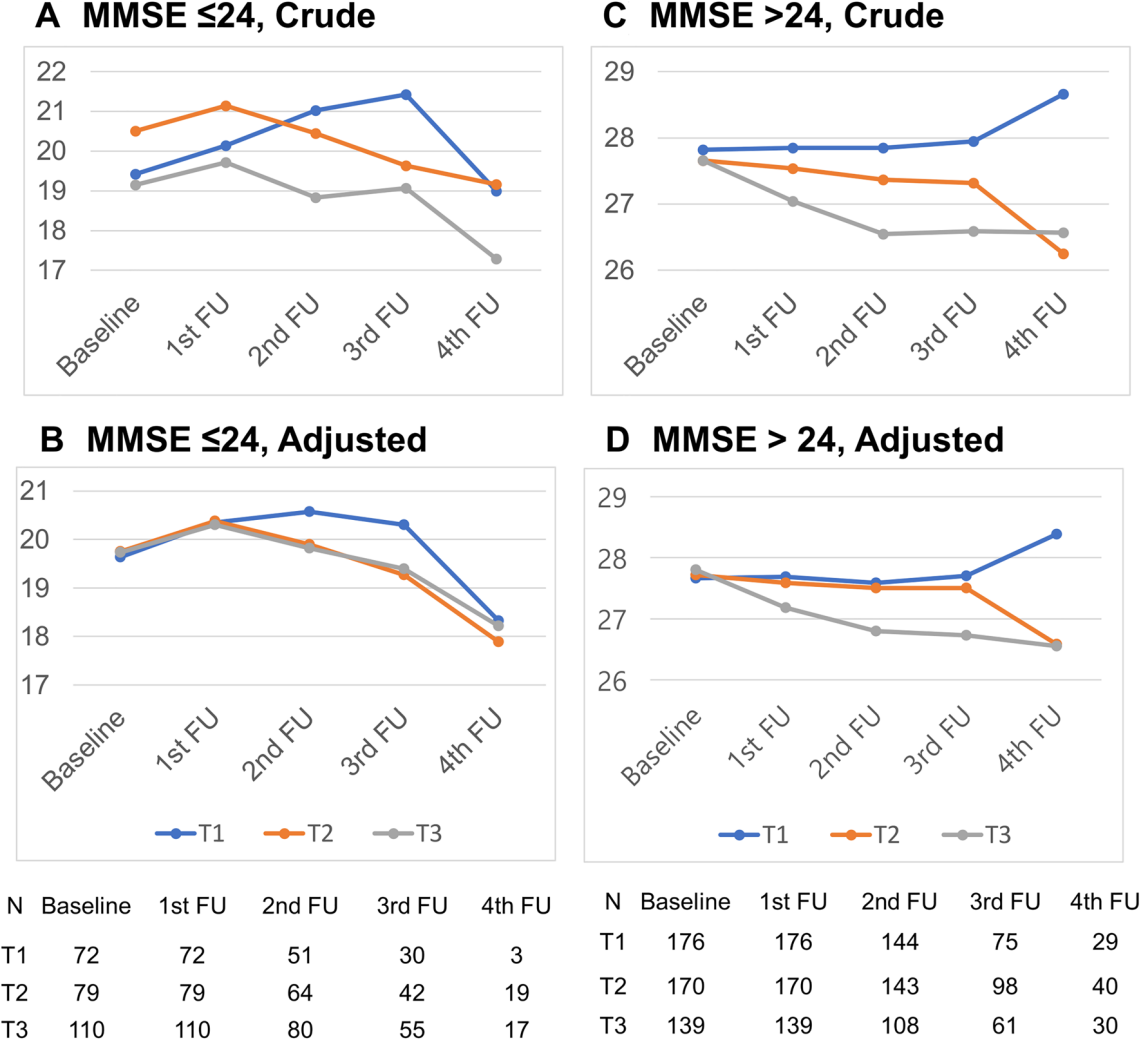
The cognition test scores from the Mixed-Model Repeated Measure analysis over the follow-up period according to initial cognitive function (MMSE ≤ 24 vs. MMSE > 24). (**A**) MMSE ≤ 24, Crude (**B**) MMSE ≤ 24, Adjusted (**C**) MMSE > 24, Crude (**D**) MMSE > 24, Adjusted. *MMSE* mini-mental state examination, *MoCA* Montreal Cognitive Assessment.

The relationship between high BPV and diagonal cognitive decline was also evident in the analysis of the subgroups stratified according to baseline cognitive status (MMSE score ≤ 24 vs. MMSE score > 24). Unlike patients with MMSE scores ≤ 24, there was a significant cognitive decline according to the BPV groups among patients with MMSE scores > 24 (*p* < 0.0001) (Fig. 3A–D and Table S6). After adjusting for initial cognitive differences between the two groups, cognitive function showed a tendency to deteriorate over time, especially in the higher BPV groups.

## Discussion

Our study showed that high BPV may be related to cognitive decline in a cohort of IS patients. This trend was also evident in the subgroups stratified according to baseline cognitive function, especially in patients with normal baseline cognitive function (MMSE score > 24). However, mean SBP was not significantly associated with cognitive decline.

Previous reports demonstrated a positive relationship between cognitive decline and high BPV^11–14^. Although the exact patho-mechanism remains unknown, there is speculation about why BPV is an independent predictor for cognitive decline. First, recurrent cerebral hypoperfusion may play a role. Cerebral blood flow remains stable when systemic BP changes within the lower and upper pressure limits of autoregulation. However, high BPV may accompany a BP below the lower limit, which results in recurrent cerebral hypoperfusion. Patients with IS have more diminished autoregulatory capacities^21,22^. Hypoperfusion may lead to selective collapse of key proteins within the paranodal axon–glial junctions that are critical in white matter function^23^. Second, BP fluctuation may contribute to amyloid-beta (Aβ) deposits and brain atrophy^23^. In healthy elderly individuals, greater sleep-systolic BPV^24,25^ and chronic hypoperfusion^26^ is associated with aggravated brain atrophy. Furthermore, transgenic mice with amyloid precursor protein mutations (TgAPP) model demonstrated that blood flow reductions promote amyloid deposition compared with wild-type mice^27^. A comorbid amyloid pathology could mediate cognitive impairment after stroke^28^. Besides cerebral hypoperfusion, endothelial dysfunction, inflammation, neurohumoral activation, and oxidative stress have been suggested as underlying mechanisms^29–31^.

It remains to be determined why mean BP was not associated with cognitive decline. Consistent with our results, although researchers observed no correlation between mean BP and dementia, an increase of 1-SD in CV was associated with a 10% increased risk of dementia^16^. Among 24,593 patients without preexisting cognitive dysfunction, the CV_SBP_ was independent predictors of cognitive decline, whereas mean SBP was not^10^. In 240 patients with Alzheimer disease, only systolic BPV showed a decrease in MMSE score^15^. Because the brain has autoregulation, a constant pressure is maintained even in patients with some degree of hypertension. Therefore, a mildly to moderately elevated BP might not affect cognitive decline. Our findings may suggest that mean BP has less impact on cognitive function than BPV.

One notable point is that BPV would have impacted cognitive decline before the index stroke. Although the study population from PICASSO was randomized, the differences in baseline cognitive function among the tertile groups might be meaningful. Since BPV does not occur at the time of the index IS, we proposed that the diminished initial cognitive status was also caused by the influence of BPV over a long period of time.

**Figure 4:**
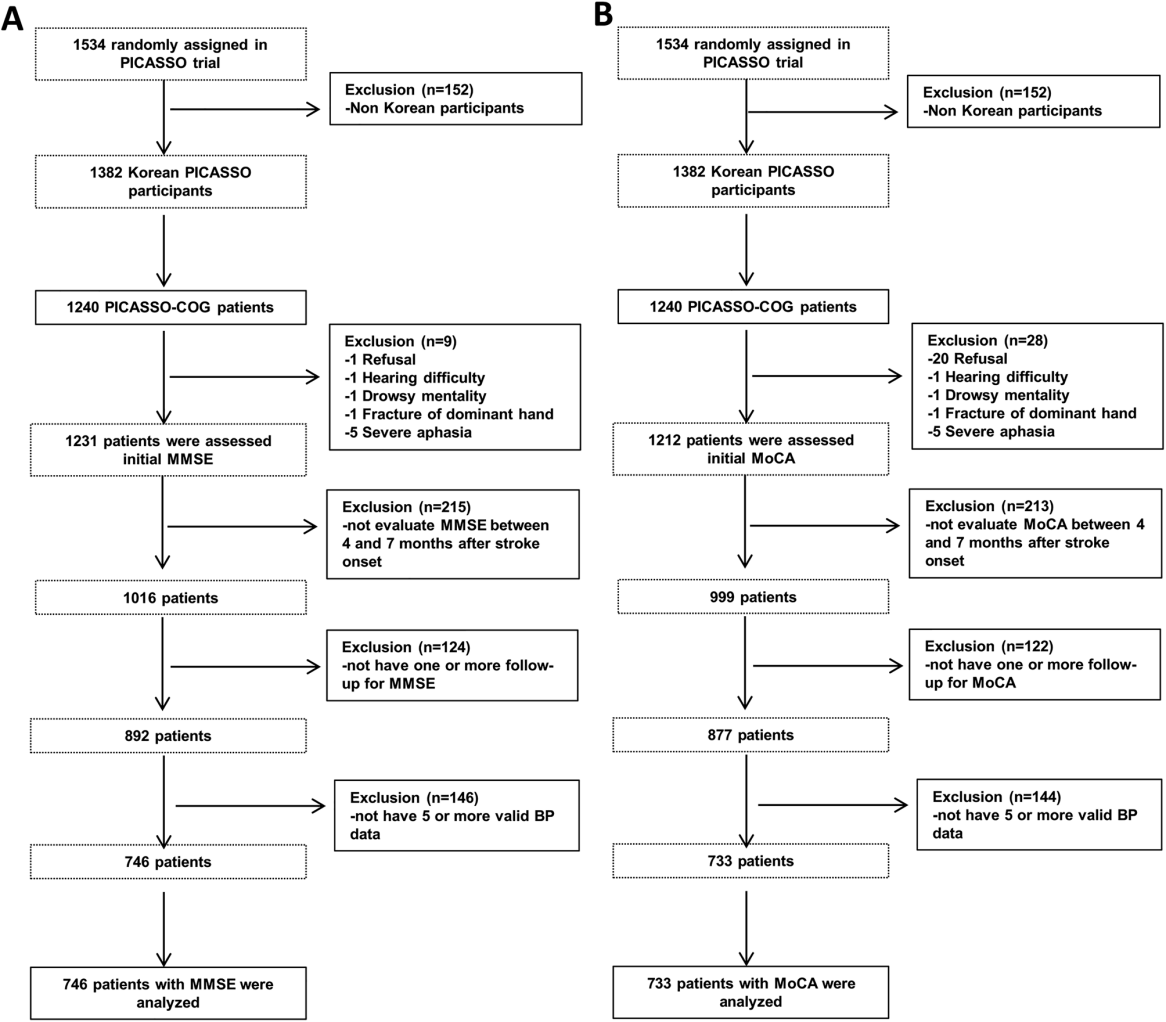
Flow chart of PICASSO-COG trial study population.

Higher BPV is an independent predictor of cognitive decline in patients without preexisting cognitive dysfunction^10,13^. To clarify it, we classified patients into two groups (MMSE score ≤ 24 vs. MMSE score > 24). It is noteworthy that high BPV was associated with significant cognitive decline in those with MMSE scores > 24 (Fig. 4). We suspect that those patients with a relatively preserved baseline cognitive function are more likely to have been influenced by BPV since they were not yet influenced by other amyloid or vascular pathologies. These findings suggest that BPV has relatively low impact on cognitive decline in patients with pathological conditions.

Our study has some specific strengths and limitations. The main study (PICASSO)^32^ had a double-blind randomized design with a large population. Second, our study utilized repeated cognition tests, whereas previous studies conducted one or two evaluations during follow-up^10,11,13^. Third, regarding methodology, compared to previous studies using linear-to-linear regression or cross-sectional methods, MMRM has been beneficially used in the analysis of longitudinal data, especially when randomly missed values are assumed^33^. Finally, we utilized both MMSE and MoCA to detect cognitive decline after stroke. In a longitudinal study, preliminary exploration using MMSE as a screening test is reasonable^15^. However, MoCA is considered a well-validated tool for screening, particularly in terms of executive function^34^. Since MoCA involves more demanding visual construction tasks, it had a lesser ceiling effect^34^.

An important limitation is that this population with multiple microbleeds and prior ICH is not necessarily representative of all IS patients. Additionally, the patients included in this study had very mild stroke severity (median NIHSS 1). Therefore, the interpretation of the results may not be generalized. Second, BP was not assessed at the same time of day for all subjects during each follow-up appointment. This limitation might contribute to the random error of BPV measurements that may attenuate the associations. Third, attrition bias may affect our results, as it was present in previous longitudinal cognitive studies^35^. Fourth, because we did not find definite evidence of a causative relationship, high BPV may be a marker of disease rather than a contributing factor. Fifth, probucol, a non-statin cholesterol-lowering agent, prevented hippocampal synaptic impairment^36^ as well as blood–brain barrier dysfunction^37^. However, to reduce the influences of probucol, we adjusted for probucol treatment in the MMRM analysis. Sixth, we did not investigate anti-hypertensive or anti-diabetic medication type, which may affect BPV. Although we have all the data on anti-hypertensive drugs, anti-diabetic drugs, and HbA1C levels, drug-types and doses are very arbitrary. Furthermore, we believe that BPV is the result of reflecting all of these factors and our aim is to evaluate the effect of BPV on cognitive decline in patients with IS. Seventh, several insights were suggested that the relationships among BPV, stroke location and cognitive impairment. However, we did not analyze those interactions in this study^38–40^. Finally, it is generally true that an increased BPV is associated with vascular events^41^. Although we evaluated the associations between BPV and stroke or composite outcomes of vascular events (Table S7), the BPV parameters showed inconsistent results. Various indices of BPV have different prognostic relevance for vascular outcomes^42^. Accordingly, a recent review suggested that BPV parameters remain a research tool rather than an index for risk stratification^43^.

Our results suggest that high BPV is closely linked to faster decline of cognitive function in a cohort of IS patients. Current treatment guidelines for high BP are based on mean values^44^. However, our findings suggest that physicians should more pay attention to BPV to prevent cognitive decline. Further investigations are required to elucidate the correlation between BPV and cognitive function.

## Methods

This study is a sub-analysis of the PICASSO trial^32^, a randomized controlled trial conducted in 67 centers in three countries (South Korea, the Philippines, and Hong Kong, China). PICASSO was a two-by-two factorial study designed to determine the efficacy and safety of cilosatzol and probucol. Between April 2010 and August 2015, this study included 1,534 patients with non-cardioembolic IS or transient ischemic attack (TIA) within 180 days, who had prior intracerebral hemorrhage (ICH) or multiple cerebral microbleeds on gradient echo imaging. Almost all patients were having significant burden of small vessel disease; approximate 70% had moderate-to-severe WMH (Fazekas scale 2–3), and the median Fazekas scale was 2. The study rationale, design, and relevant information were described previously^45^. All methods were carried out in accordance with relevant guidelines (STROBE guidelines).

The institutional review board (IRB) of each participating center approved this study, and all participants gave written informed consent (Asan medical center IRB No. 2009-0189).

### Subjects

A total of 1534 patients participated in the PICASSO trial. Among the 1382 Korean PICASSO participants, 1240 participated in the PICASSO-COG sub-study. Because neither the Mini-mental State Examination (MMSE) nor the Montreal Cognitive Assessment (MoCA) have been validated by cross-cultural studies in each language, we excluded non-Korean PICASSO participants. Detailed information about the design of the PICASSO-COG sub-study has been published previously^46^. The definitions of the baseline cognition tests and valid BP evaluations are described below. Of the PICASSO-COG participants, those for whom baseline and follow-up cognition test results were unavailable were excluded. MMSE and MoCA data for baseline and one or more follow-up tests were available for 892 and 877 patients. Among these patients, those for whom at least five valid BP readings were unavailable were excluded. Thus, the final subjects included 746 patients evaluated with MMSE and 733 analyzed using MoCA (Fig. 4A,B).

### Measurement of cognitive function

We assessed cognition at the 1-month, 4-month, annual (13, 25, 37 and 49 months after enrollment), and final visits. In patients with vascular events, we used results of the cognitive tests at the time of the event as the final cognitive outcome. To minimize the influence of acute cognitive decline by the index stroke^47^, we defined baseline cognitive tests as those conducted at 4–7 months after stroke. Baseline cognitive function was assessed at the 4-month visit after enrollment in subjects randomized within 3 months after stroke onset or at the 1-month visit after enrollment for those randomized at 4–6 months after stroke onset^46^. Cognitive tests were performed by the local investigator or the study coordinators, who were pretrained at the time of study initiation and received continued training at regular investigator meetings^46^.

### BP measurements and definition of valid blood pressure value

After randomization, participants were scheduled to visit outpatient clinics 1-month later and every 3-months thereafter that until study completion. At every scheduled and unscheduled visit, researchers at each hospital who were trained and experienced in BP measurement measured patients’ BP using an automatic sphygmomanometer with patients in a sitting position after sufficient rest. The automatic sphygmomanometer which had been calibrated in each institution was used. However, we could not validate the automatic devices for BP measurements between research centers.

We defined the first “valid” BP measurement as the BP value measured at the randomization visit when the index event was a TIA or when the time interval from the onset of the index IS to randomization was more than 30 days. When the time interval from stroke onset to randomization was 30 days or less, the first valid BP measurement was the BP level measured at the 1-month visit after randomization. By defining the valid BP this way, we minimized the influence of acute stroke on the BP value. We defined the final valid BP as the BP level measured at the closing visit when cardiovascular endpoints had not occurred. To reduce the influence of acute cardiovascular outcomes, if cardiovascular endpoints had occurred, the final valid BP was the BP value measured at the last visit before development of the endpoints. We included all BP levels measured at unscheduled and scheduled visits between the first and final valid BP measurements.

The predictive power of visit-to-visit BPV increased with more BP readings, i.e. the BPV values were not predictive if there were 4 or fewer BP readings^8^. Thus, we excluded participants for whom fewer than five valid BP measurements were available.

### Calculation of visit-to-visit BPV

We used the intra-individual standard deviation (SD) of systolic BP (SBP) as the primary parameter of BPV (BPV-SD). Although traditional measures of BPV, including the SD and coefficient of variation (CV), are often used, absolute levels of BPV are often positively correlated with mean BP levels^48^. Therefore, we calculated a transformed parameter of SD that is defined to be uncorrelated with mean BP levels: variation independent of the mean (VIM). VIM is proportional to SD/mean^x^, with x derived from curve fitting in this cohort. As a secondary parameter of BPV, we also calculated SDreg, the SD of the participant’s regression line with SBP regressed across visits^41^. Conceptually, SD is the “average” of the deviations of the mean (which is assumed to be static over time), while SDreg is the “average” of the deviations of the regression line (which assumes a linear change over time).

### Confounding variables

For each participant, we collected data on demographics, years of education, vascular risk factors, initial National Institutes of Health Stroke Scale (NIHSS) score, mean SBP, and severity of WMH on magnetic resonance imaging (MRI) using the Fazekas scale. The composite outcomes of vascular events included stroke, myocardial infarction, and vascular death.

### Statistical analysis

Baseline characteristics are expressed as number of participants (%), mean ± SD, or median (interquartile range). Participants were classified into three groups according to BPV tertiles (T1, lowest; T2, middle; and T3, highest). Differences in the distribution of baseline characteristics between the BPV tertiles were identified using the chi-square test, analysis of variance (ANOVA), or Kruskal–Wallis test as appropriate.

To compare changes in cognitive function over time between the BPV groups, a Mixed-Model Repeated Measures (MMRM) approach was used. MMRM has been extensively used in the analysis of longitudinal data, especially when missing data are a concern and some randomly missed values are assumed^33^. In this study, MMRM allowed within-subject correlation because of the repeated evaluations of cognitive test scores and different numbers of measurements among patients at the follow-up visits. The effect of different study sites was adjusted to be random in the model using an unstructured variance–covariance matrix. Subgroup analyses were then performed according to baseline cognitive function (MMSE score of > 24 vs. MMSE score of ≤ 24). Stroke or composite outcomes of vascular events were assessed using Poisson regression or the Cox proportional hazards model.

A two-sided 5% level was used to indicate statistical significance, and all statistical analyses were performed using SAS version 9.4 (SAS Institute, Cary, NC, USA).

## Supplementary Information

Supplementary Information.

## Data Availability

The datasets analysed during the present study are available from the corresponding author on reasonable request.
