# Supplementary material for: Blood pressure variability is related to faster cognitive decline in ischemic stroke patients: PICASSO subanalysis
Source: Sci Rep. 2021 Mar 3;11:5049. doi: 10.1038/s41598-021-83945-z (PMC7930263; doi:10.1038/s41598-021-83945-z)
Supplement: Supplementary file 1 — Supplementary Information. [file 41598_2021_83945_MOESM1_ESM.docx]

Supplementary materials.

**Blood Pressure Variability is Related to Faster Cognitive Decline in Ischemic Stroke Patients: PICASSO subanalysis**

**Cover title: BPV and Cognitive decline in Ischemic Stroke**

Yerim Kim, MD, PhD^1^ Jae-Sung Lim, MD, PhD^2^ Mi Sun Oh, MD, PhD ^3^ Kyung-Ho Yu, MD, PhD ^3^ Ji Sung Lee, MD, PhD ^4^ Jong-Ho Park, MD, PhD^5^ Yong-Jae Kim, MD, PhD^6^ Joung-Ho Rha, MD, PhD^7^ Yang-Ha Hwang, MD, PhD^8^ Sung Hyuk Heo, MD, PhD^9^ Seong Hwan Ahn, MD, PhD^10^ Ju-Hun Lee, MD ^1*^ Sun U. Kwon, MD, PhD ^2*^

^1^Department of Neurology, Kangdong Sacred Heart Hospital, Hallym University College of Medicine, Seoul, Republic of Korea

^2^Department of Neurology, University of Ulsan, Asan Medical Center, Seoul, Republic of Korea

^3^Department of Neurology, Hallym University Sacred Heart Hospital, Hallym University College of Medicine, Anyang, Republic of Korea

^4^Clinical Research Center, Asan Institute for Life Sciences, Asan Medical Center, University of Ulsan College of Medicine, Seoul, Republic of Korea

^5^Department of Neurology, Myongji Hospital, Hanyang University College of Medicine, Goyang, Republic of Korea

^6^Department of Neurology, Eunpyeong St. Mary’s Hospital, College of Medicine, The Catholic University of Korea, Seoul, Republic of Korea

^7^Department of Neurology, Inha University Hospital, Incheon, Republic of Korea

^8^Department of Neurology, Kyungpook National University Hospital, School of Medicine, Kyungpook National University, Daegu, Korea

^9^Department of Neurology, Kyung Hee University Medical Center, Seoul, Republic of Korea

^10^Department of Neurology, Chosun University Hospital, Gwangju, Republic of Korea

| Supplementary table 1. Comparison of clinical features between the excluded and the included patients | | | |
| --- | --- | --- | --- |
|  | Not included (n=636) | Included (n=746) | *p* value |
| Age, years (SD) | 67.8±10.4 | 64.6±10.8 | <0.001 |
| Sex, women, n(%) | 265(41.7) | 268(35.9) | 0.029 |
| Education, years | 8.0±5.0 | 8.6±4.8 | 0.014 |
| Hypertension, n(%) | 556(87.4) | 668(89.5) | 0.216 |
| Diabetes, n(%) | 211(33.2) | 231(31) | 0.38 |
| Hyperlipidemia, n(%) | 243(38.2) | 322(43.2) | 0.062 |
| Use of lipid lowering agent | 489(76.9) | 565(75.7) | 0.617 |
| Coronary artery disease, n(%) | 36(5.7) | 31(4.2) | 0.194 |
| Smoking, n(%) | 256(40.3) | 355(47.6) | 0.006 |
| Index event, n(%) |  |  | 0.318 |
| Ischemic stroke | 610(95.9) | 707(94.8) |  |
| Transient ischemic attack | 26(4.1) | 39(5.2) |  |
| Index of high risk of ICH |  |  | 0.2068 |
| Prior history of ICH | 92(18.6) | 121(16.2) |  |
| Imaging findings of ICH without clinical history | 73(14.8) | 136(18.2) |  |
| Multiple microbleeds | 329(66.6) | 489(65.5) |  |
| Baseline mean NIHSS (SD) | 2.6±3.0 | 1.8±2.0 | <0.001 |
| Baseline median NIHSS (IQR) | 1 (0 - 3) | 1 (0 - 3) | 0.0083 |
| Baseline median K-MMSE (IQR) | 25 (19 - 28) | 26 (22 - 28) | <.0001 |
| Endpoint events during follow-up |  |  |  |
| Ischemic stroke, n(%) | 65(10.2) | 23(3.1) | <0.001 |
| Intracranial hemorrhage, n(%) | 12(1.9) | 12(1.6) | 0.693 |
| All stroke, n(%) | 77(12.1) | 34(4.6) | <0.001 |
| Myocardial infarct, n(%) | 6(0.9) | 3(0.4) | 0.212 |
| Vascular death, n(%) | 4(0.6) | 6(0.8) | 0.761 |
| All death, n(%) | 31(4.9) | 17(2.3) | 0.009 |
| NIHSS: the National Institutes of Health Stroke Scale, *P*-value by Chi-square test or t-test as appropriate | | | |

Abbreviations. SD, standard deviation; NIHSS, National Institutes of Health Stroke Scale

Supplementary table 2. Cognitive scores over visits according to quartile of BPV (SBP-SDreg ) by using K-MMSE and K-MoCA

| **K-MMSE** | **BPV(SBP-SDreg) Tertile1 (ref.)** | **BPV(SBP-SDreg) Tertile 2** | **BPV(SBP-SDreg) Tertile 3** | ***p*-value**  **(a)** | ***p*-value (b)** | ***p*-value (c)** |
| --- | --- | --- | --- | --- | --- | --- |
| Initial | 25.7±4.2 | 25.0±4.8 | 24.0±4.9 | 0.0164 | 0.0001 | <.0001 |
| 1st follow-up | 26.0±4.3 | 25.1±4.8 | 23.8±5.0 |  |  |  |
| 2nd follow-up | 26.0±4.2 | 25.2±4.7 | 22.7±5.8 |  |  |  |
| 3rd follow-up | 25.6±4.6 | 24.6±5.5 | 22.5±6.1 |  |  |  |
| 4th follow-up | 25.9±4.7 | 23.7±5.8 | 19.6±5.7 |  |  |  |
| *P* for linear trend | 0.0949 | <.0001 | 0.0031 |  |  |  |
| **K-MoCA** |  |  |  |  |  |  |
| Initial | 20.5±6.0 | 19.5±6.1 | 17.7±6.4 | 0.1898 | 0.0052 | 0.0377 |
| 1st follow-up | 20.9±6.2 | 19.5±6.1 | 17.5±6.8 |  |  |  |
| 2nd follow-up | 20.9±6.1 | 19.8±6.4 | 16.3±6.9 |  |  |  |
| 3rd follow-up | 20.5±6.6 | 19.3±6.7 | 15.8±6.9 |  |  |  |
| 4th follow-up | 21.4±6.3 | 19.1±6.5 | 12.0±8.4 |  |  |  |
| *P* for linear trend | 0.2659 | 0.4591 | 0.0090 |  |  |  |

Note. The Model included BPV group, visit, BPV-by-visit interaction, age, sex, educational year, probucol(yes/no), baseline NIHSS, baseline K-MMSE or MoCA, DM, index of high risk of ICH and mean SBP as fixed effect.

Effect of sites was adjusted as random in the model.

MMRM includind BPV-by visit interaction, baseline score, and baseline score-by visit interaction

1. *p*-value by MMRM for BPV-by-visit interaction
2. *p*-value by MMRM for BPV effect
3. *p*-value by MMRM for visit effect

Abbreviations. SDreg, the SD about the participant’s regression line with SBP regressed across visits ; NIHSS, National Institutes of Health Stroke Scale; K-MMSE, Korean version mini-mental state examination; K-MoCA, Korean-Montreal Cognitive Assessment; BPV, blood pressure variability

Supplementary table 3. Cognitive scores over visits according to tertile of BPV (SBP-VIM) by using K-MMSE and K-MoCA

| **K-MMSE** | **BPV(SBP-VIM) Tertile1 (ref.)** | **BPV(SBP-VIM) Tertile 2** | **BPV(SBP-VIM) Tertile 3** | ***p*-value**  **(a)** | ***p*-value (b)** | ***p*-value (c)** |
| --- | --- | --- | --- | --- | --- | --- |
| Initial | 25.8±4.2 | 25.0±4.7 | 23.9±5.0 | 0.0008 | <.0001 | 0.0004 |
| 1st follow-up | 25.9±4.2 | 25.2±4.7 | 23.8±5.2 |  |  |  |
| 2nd follow-up | 26.1±4.3 | 25.4±4.5 | 22.9±5.7 |  |  |  |
| 3rd follow-up | 26.1±4.4 | 24.8±4.9 | 23.0±6.3 |  |  |  |
| 4th follow-up | 27.5±3.6 | 24.2±5.2 | 22.2±6.3 |  |  |  |
| *P* for linear trend | 0.3275 | <.0001 | <.0001 |  |  |  |
| **K-MoCA** |  |  |  |  |  |  |
| Initial | 20.7±5.8 | 19.3±6.4 | 17.8±6.3 | 0.0910 | 0.0228 | 0.2994 |
| 1st follow-up | 20.8±6.2 | 19.4±6.4 | 17.7±6.6 |  |  |  |
| 2nd follow-up | 21.3±5.8 | 19.7±6.4 | 16.6±6.9 |  |  |  |
| 3rd follow-up | 21.3±6.2 | 19.1±6.8 | 17.1±7.1 |  |  |  |
| 4th follow-up | 23.7±4.7 | 19.0±6.8 | 16.6±8.2 |  |  |  |
| *P* for linear trend | 0.2956 | 0.0159 | 0.0504 |  |  |  |

Note. The Model included BPV group, visit, BPV-by-visit interaction, age, sex, educational year, probucol(yes/no), baseline NIHSS, baseline K-MMSE or MoCA, DM, index of high risk of ICH and mean SBP as fixed effect.

Effect of sites was adjusted as random in the model.

MMRM includind BPV-by visit interaction, baseline score, and baseline score-by visit interaction

1. p-value by MMRM for BPV-by-visit interaction
2. p-value by MMRM for BPV effect
3. p-value by MMRM for visit effect

Abbreviations. VIM, variation independent of mean; NIHSS, National Institutes of Health Stroke Scale; K-MMSE, Korean version mini-mental state examination; K-MoCA, Korean-Montreal Cognitive Assessment; BPV, blood pressure variability

Supplementary table 4. Baseline characteristics according to tertile of mean SBP

|  | Mean SBP T1 | Mean SBP T2 | Mean SBP T3 | *p*-value |
| --- | --- | --- | --- | --- |
| Number of subjects | 249 | 248 | 249 |  |
| Age, years (SD) | 64.4±10.2 | 65.4±10.8 | 64.0±11.5 | 0.3421 |
| Sex, women, n(%) | 87(34.9) | 95(38.3) | 86(34.5) | 0.63 |
| Education, years | 8.6±4.6 | 8.8±5.0 | 8.4±4.9 | 0.6365 |
| Hypertension, n(%) | 210(84.3) | 225(90.7) | 233(93.6) | 0.0026 |
| Diabetes, n(%) | 63(25.3) | 74(29.8) | 94(37.8) | 0.0098 |
| Hyperlipidemia, n(%) | 105(42.2) | 111(44.8) | 106(42.6) | 0.8215 |
| Use of lipid lowering agent | 186(74.7) | 193(77.8) | 186(74.7) | 0.6443 |
| Coronary artery disease, n(%) | 13(5.2) | 14(5.6) | 4(1.6) | 0.0461 |
| Atrial fibrillation, n(%) | 0(0.0) | 0(0.0) | 0(0.0) |  |
| Smoking, n(%) | 133(53.4) | 110(44.4) | 112(45.0) | 0.0778 |
| Index event, n(%) |  |  |  | 0.2773 |
| Ischemic stroke | 236(94.8) | 239(96.4) | 232(93.2) |  |
| TIA | 13(5.2) | 9(3.6) | 17(6.8) |  |
| Index of high risk of ICH |  |  |  | 0.9753 |
| Prior history of ICH | 40(16.1) | 38(15.3) | 43(17.3) |  |
| Imaging findings of ICH without clinical history | 46(18.5) | 44(17.7) | 46(18.5) |  |
| Multiple microbleeds | 163(65.5) | 166(66.9) | 160(64.3) |  |
| Baseline median NIHSS (IQR) | 1 (0 - 3) | 1 (0 - 3) | 1 (0 - 3) | 0.5929 |
| Baseline median K-MMSE (IQR) | 26 (21 - 28) | 26 (24 - 29) | 26 (23 - 29) | 0.4837 |
| 24 or less, n(%) | 94(37.8) | 79(31.9) | 88(35.3) | 0.3830 |
| > 24, n(%) | 155(62.2) | 169(68.1) | 161(64.7) |  |
| Baseline median K-MoCA (IQR) | 20 (14 - 24) | 21 (17 - 24) | 21 (16 - 24) | 0.1941 |
| Treatment |  |  |  |  |
| cilosstazol, n(%) | 142(57.0) | 119(48.0) | 120(48.2) | 0.0704 |
| probucol, n(%) | 139(55.8) | 127(51.2) | 109(43.8) | 0.0252 |
| Mean SBP, mmHg (SD) | 124.3±15.2 | 132.0±14.9 | 145.3±16.7 | <.0001 |
| Mean DBP, mmHg (SD) | 75.0±9.7 | 78.2±10.7 | 84.2±12.6 | <.0001 |
| Median BP readings, (IQR) | 11 (8 - 14) | 12 (9 - 14) | 10 (8 - 14) | 0.1202 |
| Median FU periods, (IQR), unit: years | 3 (2 - 3) | 3 (2 - 3) | 3 (2 - 3) | 0.1087 |
| Baseline WMH, n(%) |  |  |  |  |
| severe (Fazeka score =3) | 68(28.8) | 61(25.4) | 52(21.7) | 0.1998 |

*P*-value by Chi-square test, ANOVA or Kruskal-Wallis test as appropriate

Abbreviations. SD, standard deviation; NIHSS, National Institutes of Health Stroke Scale; K-MMSE, Korean version mini-mental state examination; K-MoCA, Korean-Montreal Cognitive Assessment; IQR, interquartile range; SBP, systolic blood pressure; DBP, diastolic blood pressure; WMH, whitematter hyperintensity

Supplementary table 5. Cognitive scores over visits according to tertile of mean SBP by using K-MMSE and K-MoCA

| **K-MMSE** | **Mean SBP Tertile 1 (ref.)** | **Mean SBP Tertile 2** | **Mean SBP Tertile 3** | **p-value**  **(a)** | **p-value (b)** | **p-value (c)** |
| --- | --- | --- | --- | --- | --- | --- |
| Initial | 24.5±5.1 | 25.1±4.5 | 25.1±4.5 | 0.9535 | 0.1330 | 0.0003 |
| 1st follow-up | 24.5±5.2 | 25.2±4.5 | 25.3±4.6 |  |  |  |
| 2nd follow-up | 24.5±5.3 | 25.1±4.7 | 25.0±5.1 |  |  |  |
| 3rd follow-up | 24.2±6.0 | 25.1±4.8 | 24.8±5.1 |  |  |  |
| 4th follow-up | 24.2±5.9 | 25.5±4.8 | 23.8±5.7 |  |  |  |
| P for linear trend | 0.0011 | 0.0317 | 0.0268 |  |  |  |
| **K-MoCA** |  |  |  |  |  |  |
| Initial | 18.6±6.7 | 19.7±6.1 | 19.5±6.0 | 0.7812 | 0.6435 | 0.2083 |
| 1st follow-up | 18.7±7.0 | 19.6±6.2 | 19.6±6.3 |  |  |  |
| 2nd follow-up | 18.6±7.1 | 19.8±6.3 | 19.6±6.5 |  |  |  |
| 3rd follow-up | 18.5±7.7 | 19.9±6.3 | 19.2±6.6 |  |  |  |
| 4th follow-up | 20.0±7.4 | 20.4±6.6 | 18.3±7.6 |  |  |  |
| P for linear trend | 0.5520 | 0.2306 | 0.0602 |  |  |  |

Note. The Model included BPV group, visit, BPV-by-visit interaction, age, sex, educational year, probucol treatment, baseline NIHSS, baseline K-MMSE or K-MoCA, DM, index of high risk of ICH and SBP-SD as fixed effect.

Effect of sites was adjusted as random in the model.

MMRM including BPV-by visit interaction, baseline score, and baseline score-by visit interaction

1. p-value by MMRM for BPV-by-visit interaction
2. p-value by MMRM for BPV effect
3. p-value by MMRM for visit effect

Abbreviations. SBP, systolic blood pressure; K-MMSE, Korean version mini-mental state examination; K-MoCA, Korean-Montreal Cognitive Assessment; MMRM, Mixed-Model Repeated Measures; BPV, blood pressure variability; SD, standard deviation

Supplementary table 6. Cognitive scores over visits according to tertile of BPV (SBP-SD) depending on the baseline cognitive status (K-MMSE ≤24 versus K-MMSE>24)

| **K-MMSE**≤24 | **BPV Tertile 1 (ref.)** | **BPV Tertile 2** | **BPV Tertile 3** | **p-value**  **(a)** | **p-value (b)** | **p-value (c)** |
| --- | --- | --- | --- | --- | --- | --- |
| Initial | 19.4±4.6 | 20.5±2.9 | 19.1±4.1 | 0.8364 | 0.6232 | 0.0012 |
| 1st follow-up | 20.1±5.0 | 21.1±4.2 | 19.7±5.1 |  |  |  |
| 2nd follow-up | 21.0±5.3 | 20.5±4.4 | 18.8±5.6 |  |  |  |
| 3rd follow-up | 21.4±4.7 | 19.6±4.8 | 19.1±6.4 |  |  |  |
| 4th follow-up | 19.0±11.8 | 19.2±5.6 | 17.3±4.7 |  |  |  |
| P for linear trend | 0.4163 | 0.0014 | 0.0092 |  |  |  |
| **K-MMSE**>25 |  |  |  |  |  |  |
| Initial | 27.8±1.7 | 27.7±1.6 | 27.7±1.7 | <.0001 | <.0001 | 0.0006 |
| 1st follow-up | 27.9±2.0 | 27.5±2.1 | 27.0±2.2 |  |  |  |
| 2nd follow-up | 27.8±2.1 | 27.4±2.2 | 26.5±3.1 |  |  |  |
| 3rd follow-up | 27.9±2.4 | 27.3±2.8 | 26.6±2.6 |  |  |  |
| 4th follow-up | 28.7±1.4 | 26.3±2.6 | 26.6±3.3 |  |  |  |
| P for linear trend | 0.0502 | 0.0004 | <.0001 |  |  |  |

Note. The Model included BPV group, visit, BPV-by-visit interaction, age, sex, educational year, probucol treatment, baseline NIHSS, baseline K-MMSE or K-MoCA, DM, index of high risk of ICH and mean SBP as fixed effect.

Effect of sites was adjusted as random in the model.

MMRM including BPV-by visit interaction, baseline score, and baseline score-by visit interaction

1. p-value by MMRM for BPV-by-visit interaction
2. p-value by MMRM for BPV effect
3. p-value by MMRM for visit effect

Abbreviations. SBP, systolic blood pressure; K-MMSE, Korean version mini-mental state examination; K-MoCA, Korean-Montreal Cognitive Assessment; MMRM, Mixed-Model Repeated Measures; BPV, blood pressure variability; SD, standard deviation

Supplementary table 7. Incidence rates of stroke or composite outcomes of vascular events according to tertile of BPV (SBP-SD, SBP-SDreg and SBP-VIM) by using K-MMSE and K-MoCA

|  | **K-MMSE** | | | *p*-value | **K-MoCA** | | | *p*-value |
| --- | --- | --- | --- | --- | --- | --- | --- | --- |
| **SBP-SD** | SBP-SD  Tertile1 (ref.) | SBP-SD  Tertile 2 | SBP-SD  Tertile 3 |  | SBP-SD Tertile1 (ref.) | SBP-SD  Tertile 2 | SBP-SD  Tertile 3 |  |
| **Poisson regression** |  |  |  |  |  |  |  |  |
| Composite outcomes of vascular events | 2.28  (1.35-3.85) | 2.29  (1.41-3.75) | 1.81  (1.03-3.18) | 0.7864 | 2.32  (1.38-3.92) | 2.18  (1.32-3.62) | 1.69  (0.94-3.06) | 0.7150 |
| Stroke | 1.95  (1.11-3.44) | 1.72  (0.98-3.03) | 1.50  (0.81-2.80) | 0.8293 | 1.99  (1.13-3.51) | 1.60  (0.89-2.89) | 1.39  (0.72-2.66) | 0.7030 |
| **Cox proportional hazards model** |  |  |  |  |  |  |  |  |
| Composite outcomes of vascular events | 1 | 0.96  (0.47-1.97) | 0.77  (0.36-1.67) | 0.7835 | 1 | 0.89  (0.43-1.85) | 0.71  (0.32-1.56) | 0.6889 |
| Stroke | 1 | 0.84  (0.38-1.87) | 0.75  (0.32-1.74) | 0.7948 | 1 | 0.76  (0.34-1.73) | 0.67  (0.28-1.60) | 0.6450 |
| **SBP-SDreg** | SBP-SDreg  Tertile1 (ref.) | SBP-SDreg  Tertile 2 | SBP-SDreg  Tertile 3 |  | SBP-SDreg  Tertile1 (ref.) | SBP-SDreg  Tertile 2 | SBP-SDreg  Tertile 3 |  |
| **Poisson regression** |  |  |  |  |  |  |  |  |
| Composite outcomes of vascular events | 0.92  (0.44-1.93) | 1.77  (1.00-3.11) | 4.29  (2.85-6.46) | 0.0005 | 0.94  (0.45-1.97) | 1.81  (1.03-3.18) | 3.98  (2.60-6.11) | 0.0019 |
| Stroke | 0.66  (0.27-1.58) | 1.32  (0.69-2.55) | 3.73  (2.41-5.78) | 0.0005 | 0.67  (0.28-1.61) | 1.35  (0.70-2.60) | 3.41  (2.15-5.42) | 0.0019 |
| **Cox proportional hazards model** |  |  |  |  |  |  |  |  |
| Composite outcomes of vascular events | 1 | 2.01  (0.79-5.11) | 5.83  (2.47-13.77) | <.0001 | 1 | 2.03  (0.80-5.16) | 5.35  (2.24-12.75) | 0.0002 |
| Stroke | 1 | 2.12  (0.71-6.33) | 7.27  (2.68-19.73) | <.0001 | 1 | 2.14  (0.71-6.41) | 6.59  (2.40-18.08) | 0.0003 |
| **SBP-VIM** | SBP-VIM  Tertile1 (ref.) | SBP-VIM  Tertile 2 | SBP-VIM  Tertile 3 |  | SBP-VIM  Tertile1 (ref.) | SBP-VIM  Tertile 2 | SBP-VIM  Tertile 3 |  |
| **Poisson regression** |  |  |  |  |  |  |  |  |
| Composite outcomes of vascular events | 1.57  (0.85-2.92) | 1.99  (1.18-3.36) | 2.83  (1.78-4.49) | 0.3025 | 1.61  (0.87-2.99) | 2.02  (1.19-3.41) | 2.57  (1.57-4.19) | 0.4998 |
| Stroke | 1.26  (0.63-2.52) | 1.28  (0.67-2.46) | 2.67  (1.66-4.29) | 0.0956 | 1.29  (0.64-2.57) | 1.30  (0.67-2.49) | 2.41  (1.45-4.00) | 0.2127 |
| **Cox proportional hazards model** |  |  |  |  |  |  |  |  |
| Composite outcomes of vascular events | 1 | 1.20  (0.53-2.71) | 1.81  (0.83-3.91) | 0.2716 | 1 | 1.18  (0.53-2.67) | 1.60  (0.73-3.53) | 0.4745 |
| Stroke | 1 | 0.96  (0.37-2.50) | 2.14  (0.92-4.95) | 0.0761 | 1 | 0.95  (0.37-2.46) | 1.88  (0.80-4.43) | 0.1780 |

Note. Poisson regression incidence: per 100 person-years (95% confidence interval); Cox proportional hazards model: hazard ration (95% confidence interval)

Abbreviations. SD, standard deviation; MMSE, Mini-mental state examination; MoCA, Montreal Cognitive Assessment
